# Controlled Synthesis of Ultrathin Sb_2_Se_3_ Nanowires and Application for Flexible Photodetectors

**DOI:** 10.1002/advs.201500109

**Published:** 2015-06-25

**Authors:** Guihuan Chen, Wenliang Wang, Chunde Wang, Tao Ding, Qing Yang

**Affiliations:** ^1^ Hefei National Laboratory for Physical Sciences at Microscale (HFNL)Department of ChemistryLaboratory of Nanomaterials for Energy Conversion (LNEC)University of Science and Technology of China (USTC)HefeiAnhui230026P. R. China

**Keywords:** antimony selenide, flexile photodetector, high‐performance photoresponse, urtrathin nanowires

## Abstract

**A new solvothermal approach** is introduced to synthesize ultrathin Sb_2_Se_3_ nanowires with diameters ranging from 10 to 20 nm and with length up to 30 μm. The Sb_2_Se_3_ nanowire‐based photodetectors are firstly fabricated on polyethylene terephthalate and printing paper substrates, which exhibit excellent response to visible light with fast response time (0.18 and 0.22 s), high flexibility, and durability.

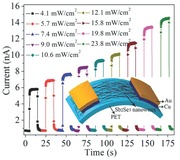

A new solvothermal approach is introduced to synthesize ultrathin Sb_2_Se_3_ nanowires with diameters ranging from 10 to 20 nm and with length up to 30 μm. The Sb_2_Se_3_ nanowire‐based photodetectors are firstly fabricated on polyethylene terephthalate (PET) and ordinary printing paper substrates, which exhibit excellent response to visible light with fast response time (0.18 and 0.22 s), high flexibility and durability.

1D semiconductor nanowires have been an exciting research field due to their potential application in artificial photosynthesis, optical wavelength filters, photovoltaic technology, electronic and optoelectronic devices.^1^, ^2^, ^3^, ^4^, ^5^ In the past decades, tremendous progress has been made in this field and much insight has been gained on tuning their electrical and optical properties by controlling their sizes and dimensions.^6^ It is known that nanowires with high aspect ratios can provide a low‐scattering transport path of the carriers and the electron and hole energy states become discrete due to the spatial confinement, which are highly beneficial for achieving high‐performance electrical or optical properties.^7^ Otherwise, nanowires are of excellent mechanical flexibility which is easy to satisfy the requirements of portability and mechanical stability in flexile optoelectronic devices.^8^, ^9^

Antimony selenide (Sb_2_Se_3_) is a typical narrow band semiconductor with a good chemical stability and ≈1.21 eV band gap,^10^ which make it an ideal candidate for visible–near‐infrared photodetectors. Zhao et al. reported a microwave‐assisted synthesis of Sb_2_Se_3_ nanorods with diameters ranging from 40 to 70 nm and lengths in the range of 400–1200 nm. Yang et al. obtained the Sb_2_Se_3_ nanorods with diameters between 40 and 200 nm via a vapor–liquid–solid approach. Wang et al. developed a novel gas‐induced‐reduction method to obtain the Sb_2_Se_3_ nanorods with diameters of ≈200 nm and lengths of 3–5 μm at 200 °C. Ma et al. prepared Sb_2_Se_3_ nanowires with diameters of 28–34 nm and length of several hundred nanometers via a solvothermal method.^11^, ^12^, ^13^, ^14^ However, to the best of our knowledge, there is no report on the controllable synthesis of Sb_2_Se_3_ ultrathin nanowires with diameters less than 20 nm. In the present work, an alternative organometallic synthetic route is developed for the growth of ultrathin Sb_2_Se_3_ nanowires with high quality and ultrahigh aspect ratios from the reaction of triphenylantimony with dibenzyldiselenide in ethanol at 200 °C using oleylamine and polyvinylpyrrolidone (PVP) as surfactants. The diameters of the Sb_2_Se_3_ nanowires are mainly ranging from 10 to 20 nm with the length up to 30 μm. Meanwhile, the as‐obtained ultrathin Sb_2_Se_3_ nanowires have complementary absorption spectra in ultraviolet, visible and near‐infrared spectrum, which suggests potential application in the photoelectric devices.

Flexible devices have attracted increasing attention due to their potential applications in outfitting robots, medical prostheses, future paper displays, liquid crystal displays and wearable energy harvesting devices.^15^, ^16^, ^17^, ^18^, ^19^ Recently, extensive research is actively focused on flexible photodetectors, which have important applications in wearable communication devices, sensory skins for robotics, biomedicine, aerospace science and civil engineering that need special requirements of flexibility, lightweight, construction in thin and mechanical stability.^20^, ^21^, ^22^ Zhai et al. fabricated the Sb_2_Se_3_ single‐nanowire photodetector on SiO_2_/Si substrate with Ti/Au (10 nm/100 nm) electrodes and the device exhibited an on/off ratio of 15 with rise and decay times of 0.3/0.3 s under 615 nm illumination (1.68 mW cm^−2^). Choi et al. also fabricated the Sb_2_Se_3_ single‐nanowire photodetector on SiO_2_/Si substrate with Ti/Au (20 nm/100 nm) electrodes and the on/off ratio of the device was found to be 75 under 655 nm illumination (15 mW cm^−2^). Liu et al. fabricated the Sb_2_Se_3_ nanowire photodetector on SiO_2_/Si substrate with Au electrodes, and the device displayed an on/off ratio of 150 and response/recovery times of 0.2/1.2 s under a xenon lamp (14.4 mW cm^−2^).^23^, ^24^, ^25^ However, Sb_2_Se_3_ nanowire photodetectors based on flexile substrates have not been studied up to date. Herein, we report a simple fabrication of flexible photodetectors that are successfully fabricated by depositing Sb_2_Se_3_ nanowires on flexible ordinary printing paper in addition to PET substrates. The as‐fabricated photodetector based on PET exhibits an on/off photocurrent ratio of 19 at 23.8 mW cm^−2^ with fast response and recover time of 0.18/0.20 s. The flexile photodetector fabricated on printing paper displays an on/off photocurrent ratio of 16 at 31.3 mW cm^−2^ with fast response and recover time of 0.22/0.25 s. Moreover, the two photo­detectors exhibit high flexibility and mechanical stability. The measured results imply that the ultrathin Sb_2_Se_3_ nanowires are considerably potential for the application of versatile, low‐cost, flexible and portable electronic and optoelectronic devices.

**Figure** **1**a shows a typical X‐ray powder diffraction (XRD) pattern of the as‐prepared Sb_2_Se_3_ nanowires (top) and the corresponding standard Joint Committee on Powder Diffraction Standards (JCPDS) card No. 72‐1184 (bottom), which indicates that the products are highly pure orthorhombic phase with space group of *P*_bnm_. Figure 1b demonstrates the scheme of Sb_2_Se_3_ crystal structure displaying a perspective of the (001) facet. It can be observed that the structure of Sb_2_Se_3_ consists of infinitely long ribbon‐like [Sb_4_Se_6_]*_n_* units extending along c‐axis. These 1D ribbons coordinate with each other by intermolecular attraction, creating distinct layers along the [100] and [010] direction.^23^, ^26^ The low‐magnification SEM in Figure 1c shows the general morphology of the Sb_2_Se_3_ nanowires, from which one can see that the length of the nanowires are up to 30 μm. A high‐magnification SEM (Figure 1d) further reveals that the products are composed of a large number of nanowires with quite uniform diameters throughout their axial direction. The TEM image (Figure 1e) demonstrates that the surface of nano­wires is smooth and their diameters are mainly ranging from 10 to 20 nm, which could be observed from the histogram of the diameter distribution (inset in Figure 1e). Figure 1f exhibits the lattice fringes, where the plane spacings of 0.343 nm and 0.317 nm correspond to the lattice planes of (111) and (230) facets, respectively. These results indicate that the preferential growth occurs along the [001] direction, which is coincident with that of the Sb_2_Se_3_ nanorods reported previously.^11^, ^24^, ^27^ In addition, Raman and XPS measurements have been performed to reveal the compositional and chemical state of the products (Figure S1, Supporting Information). In the Raman spectrum (Figure S1a, Supporting Information), the peaks at 124, 189, 208 and 253 cm^−1^ are coincident with the previous investigations for the Sb_2_Se_3_ nanostructures.^23^, ^28^ The XPS results further demonstrate that both Sb and Se elements are in their expected oxidation states (Figure S1b‐d, Supporting Information).^29^

**Figure 1:**
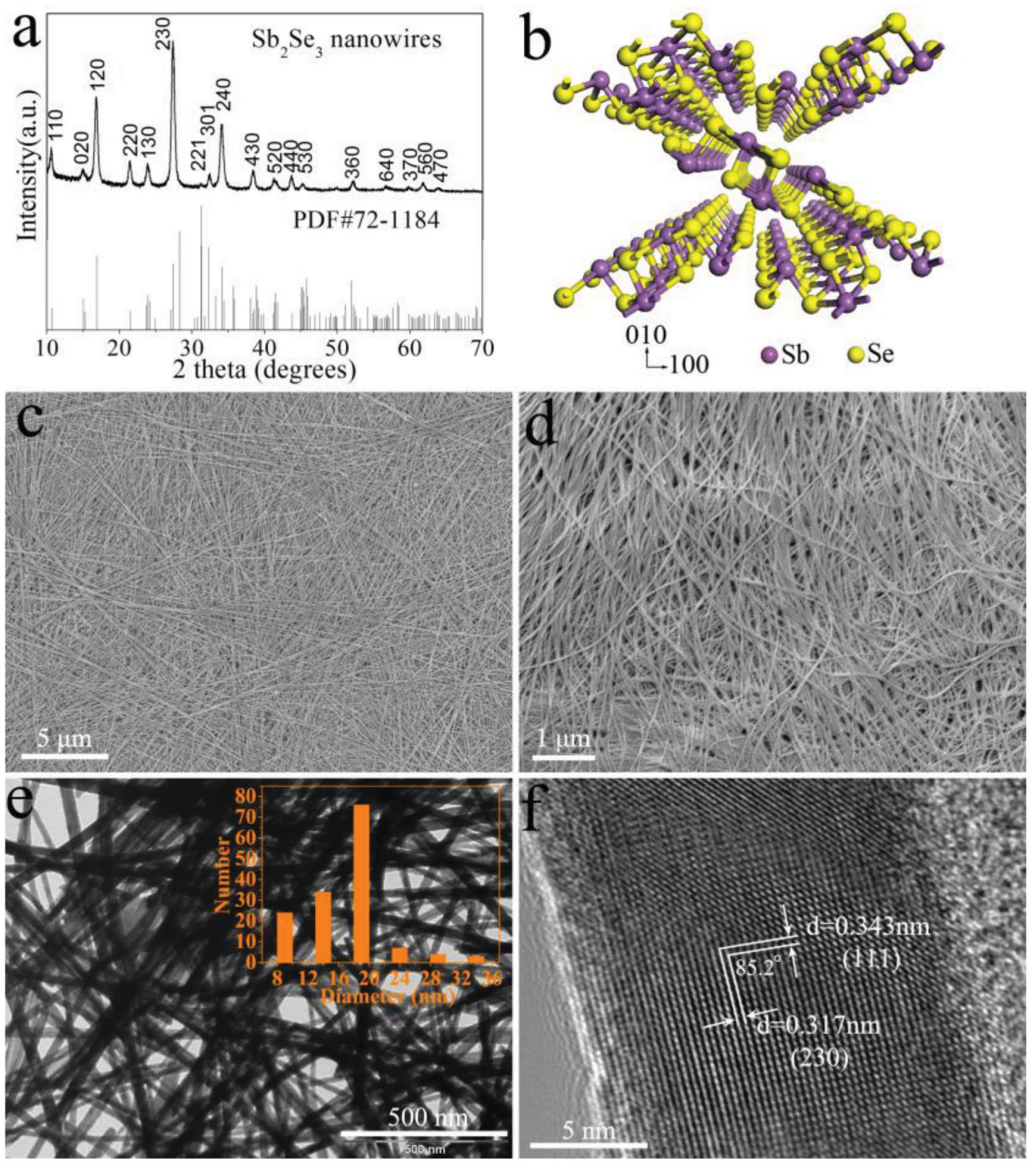
a) XRD pattern of products synthesized via reaction of triphenylantimony with dibenzyldiselenide in ethanol at 200 °C for 16 h (top) and JCPDS card, No. 72‐1184 (down). b) The scheme of crystal structure of the Sb_2_Se_3_ for the perspective of (001) facet, c) low‐ and d) high‐magnification SEM. e) TEM and histogram of the nanowires diameter distribution (inset) and f) HR‐TEM images of the nanowire.

**Figure** **2** displays the SEM and TEM images of the products obtained from different reaction temperature with other conditions unchanging. The corresponding XRD patterns are displayed in Figure S2 (Supporting Information), indicating the pure crystalline Sb_2_Se_3_ products. When the reaction temperature is set at 180 ºC, the products are composed of uniform straw‐like sheaves and the individual sheaf measures about 20 μm in length (Figure 2a). From the TEM image in Figure 2b, it can be observed that the symmetric sheaf‐like structures are assembled by the well‐aligned ultrathin nanowires with two fantails oriented to the center and the nanowires have uniform diameters of ∼20 nm (Figure 2c). Figure 2d shows the scanning electron microscopy (SEM) image of the products obtained at 220 °C. The products are discrete nanowires with the length up to 30 μm but the diameters of the nano­wires seem not very uniform. As demonstrated in Figure 2e,f, the diameters of the nanowires are ranging from 30 to 80 nm, indicating that the higher temperature has made the diameters of the nanowires increased. It is noticeable that oleylamine and PVP are used as surfactants in the synthesis process. Experiments reveal that both oleylamine and PVP play critical roles in the formation of ultrathin Sb_2_Se_3_ nano­wires. Figure S3a,b (Supporting Information) demonstrates that the resulted samples in the absence of oleylamine and PVP are composed of many 3D hierarchical architectures assembled by many 1D Sb_2_Se_3_ subunits (with diameters of 200–500 nm) and some rod‐like Sb_2_Se_3_ nanostructures (with diameters of 500–1000 nm). As shown in Figure S3c,d (Supporting Information), the products synthesized without PVP consist of orthorhombic Sb_2_Se_3_ nanorods with diameters of 200–500 nm and Sb_2_Se_3_ microtubes with diameters up to 1.5 μm. The obtained products synthesized without oleylamine are mainly Sb_2_Se_3_ nanorods with an average diameter of 500 nm (Figure S3e,f, Supporting Information). Above results indicate that the ultrathin Sb_2_Se_3_ nanowires can be selectively prepared by rationally tuning the temperature with PVP and oleylamine as surfactants. A Fourier transformed infrared (FT‐IR) spectrum of the as‐obtained products (Figure S4, Supporting Information) confirms the effects of oleylamine and PVP on the growth of the ultrathin Sb_2_Se_3_ nanowires. In addition, it is revealed that the ultrathin Sb_2_Se_3_ nanowires exhibit some different optical properties as compared to bulk Sb_2_Se_3_ due to the quantum effect (Figure S5, Supporting Information).

**Figure 2:**
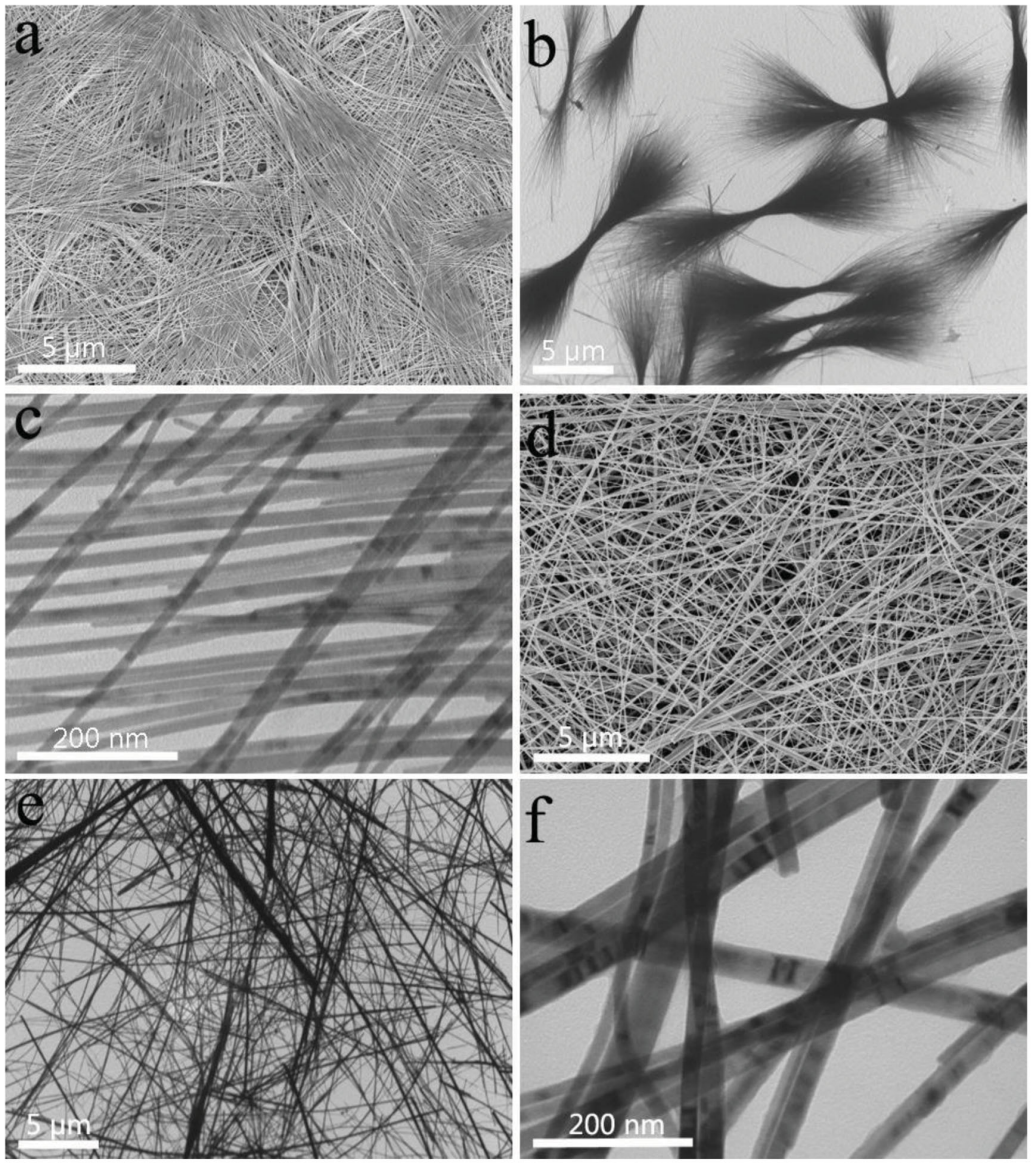
SEM and TEM images of the products synthesized at different temperature while other conditions unchanging: a) SEM, b) low‐ and c) high‐magnification TEM of the samples synthesized at 180 °C, d) SEM, e) low‐ and f) high‐magnification TEM of the samples synthesized at 220 °C.

**Figure** **3**a demonstrates the photograph (top) and schematic illustration (bottom) of the photodetector fabricated on a flexible plastics substrate of PET. Figure 3b plots the current‐voltage (*I–V*) curves of the device measured under illumination and dark conditions, respectively. The linear curves indicate the good ohmic contacts between the Cu/Au electrodes and the Sb_2_Se_3_ nanowires which are favorable for electron transmission. Figure 3c shows the time response of the device under irradiation with light intensities from 4.1 to 23.8 mW cm^−2^ at a voltage of 5.0 V, giving an on/off ratio of 19 (on/off current of 14.76/0.78 nA) at 23.8 mW cm^−2^. It can be observed that the current increases with increasing light intensity and no typical saturation behavior exists due to the low density of the incident light, which is consistent with the fact that the charge‐carrier photogeneration efficiency is proportional to the absorbed photon flux. The corresponding light‐intensity dependence of the photocurrent is demonstrated in Figure 3d, which can be expressed as a power law of *I_P_* ≈ *P^θ^*, where *I*_P_, *P*, and *θ* represent the photocurrent, light power intensity, and the response of the photocurrent to light intensity, respectively. Here the measurement results reveal a power dependence of *I_P_* ≈ *P* ^0.54^. According to previous reports, the nonunity exponent may result from the complex process of electron–hole generation, recombination, and trapping within a semiconductor, which will lead to the final saturation of the photocurrent as the light intensity increases.^30^, ^31^ Otherwise, the photo­response switching behaviors of the device are measured by periodically turning on and off the light (23.8 mW cm^−2^) at the voltage of 1.0, 5.0 and 10.0 V (Figure S6, Supporting Information), indicating the high stability of the flexile photodetector. It is well known that response and recovery time are critical parameters to evaluate the performance of a photodetector which are defined as the time required for dark current to increase to 90% of the peak value of photocurrent or vice versa. From one period of light excitation as shown in Figure 3e, the response and recovery time are determined to be 0.18 and 0.20 s, respectively, which is faster than that of previous reported Sb_2_Se_3_ photodetectors and many other flexile nanowire photodetectors.^23^, ^24^, ^32^, ^33^ The fast response time of the device may result from the high quality of the Sb_2_Se_3_ nanowires and good ohmic contacts with no interfacial barrier or traps formed between the ultrathin Sb_2_Se_3_ nanowires and the Cu/Au electrodes. Moreover, flexible optoelectronics are required to withstand varied mechanical deformations, so the electrical performance of the device at different bending conditions is measured. Figure 3f depicts the *I–V* characteristics of the flexile photodetector at different bending angles or twisting positions, where the positive and negative values are defined according to the folding direction of the device as demonstrated in the inset of Figure 3f. It is revealed that the photocurrent of the device is almost invariant of bending states. To further investigate the mechanical stability of the device, the photocurrent is checked under different bending conditions, as shown in Figure S7 (Supporting Information). The photocurrent is nearly unchanged in varied bending states, demonstrating the high flexibility and durability of the flexile photodetector.

**Figure 3:**
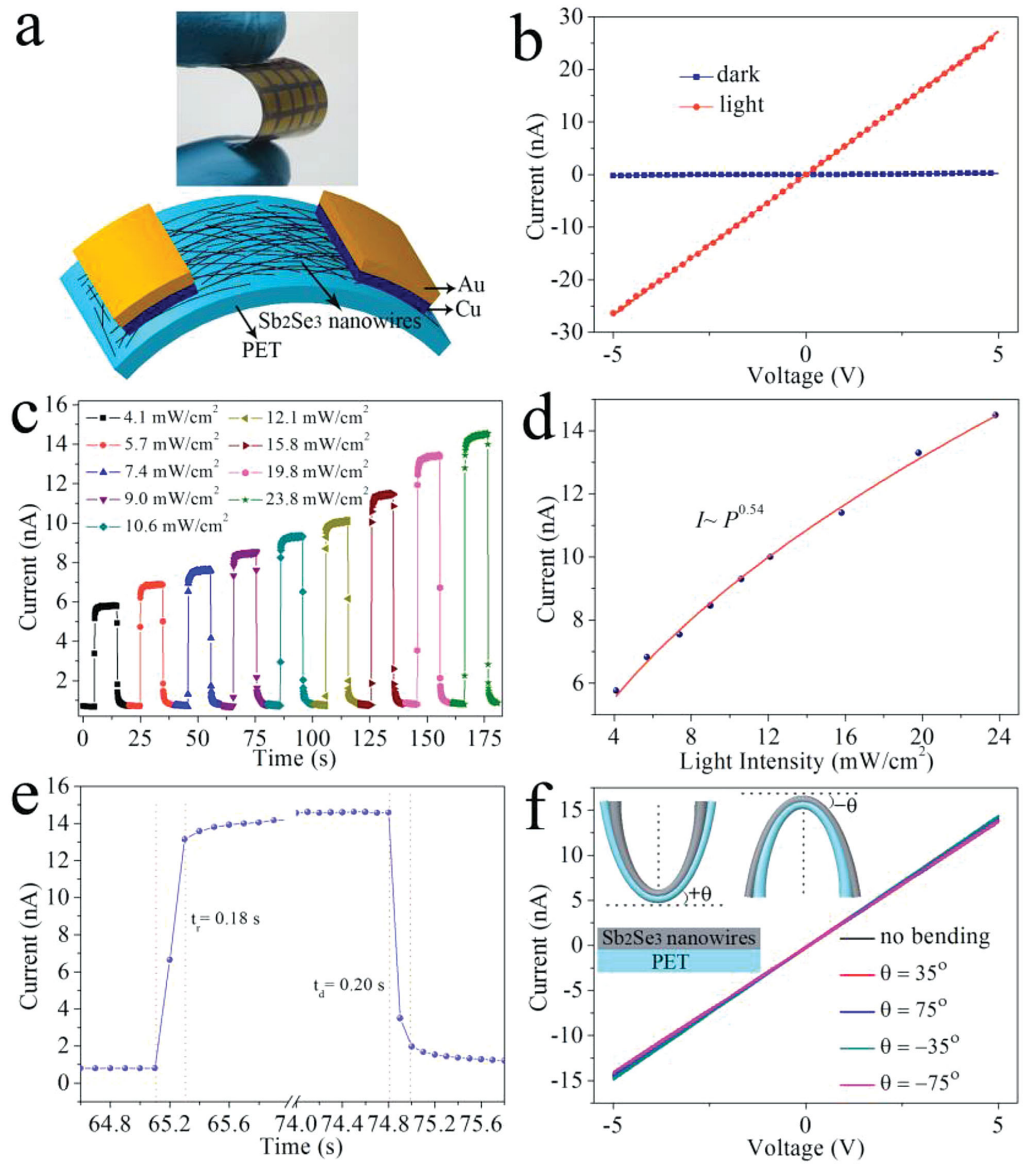
The photoresponsive characteristics of the flexile Sb_2_Se_3_ photodetector fabricated on PET substrate: a) photograph of the constructed device (top) and the schematic illustration of a flexible photodetector (bottom), b) the *I–V* curves of the device measured in the dark and under light, c) the time function of the on–off photocurrent response of the device at different light intensity at a voltage of 5.0 V, d) the photocurrent measured as a function of incident light at a voltage of 5.0 V, e) photocurrent measured in one period of modulation of the light intensity demonstrating the response and recovery time of the photodetector, and f) *I–V* curves measured at different bending angles. Inset: Schematic of the bending directions and bending angle.

The photocurrent performances of the photodetector fabricated on an ordinary printing paper are summarized in **Figure** **4**. Figure 4a shows typical *I–V* characteristics of the devices in the dark and under illumination, indicating good ohmic contacts between the Sb_2_Se_3_ nanowires and Cu/Au electrodes. The inset of Figure 4a is the photograph of the final device fabricated on the printing paper. Figure 4b shows the time responses under light intensities from 4.1 to 31.3 mW cm^−2^ at 5.0 V. It can be observed that the current increases with increasing light intensity with an on/off ratio of 16 (on/off current of 3.53/0.22 nA) at 31.3 mW cm^−2^, demonstrating a power dependence of *I ≈ P* ^0.44^ (Figure S8, Supporting Information). As demonstrated in Figure 4c, the response and recovery time are determined to be 0.22 and 0.25 s, respectively, which are equivalent to that of the photodetector fabricated on the PET substrate. Figure 4d depicts the time‐dependent measurement of the on–off photocurrent response before and after bending for 100 times with a bending angle about 75°. Notably, the photocurrent and on/off switching ratio are almost invariant of bending, suggesting the high mechanical stability of the device. To further demonstrate the flexibility of the detector based on printing paper, the optoelectronic performance of the device when bent to different angles has been precisely assessed under constant light intensity (as shown in the inset of Figure S9, Supporting Information). The minor change of the *I–V* curves in Figure S9 (Supporting Information) ensures the high flexibility and durability of the device. The measurement results demonstrate significant potential of the ultrathin Sb_2_Se_3_ nanowires for the development of versatile, low‐cost, flexible, and portable electronic and optoelectronic devices.

**Figure 4:**
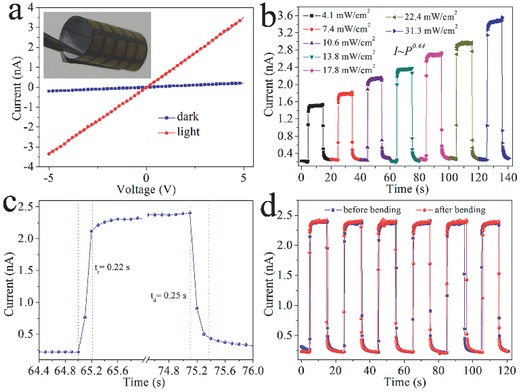
The photoresponsive characteristics of the flexile Sb_2_Se_3_ photodetector fabricated on printing paper substrate: a) the *I–V* curves of the device in the dark and light (inset showing the photograph of the flexile photodetectors based on ordinary printing paper), b) the time function of the on‐off photocurrent response of the device at different light intensity at a voltage of 5.0 V, c) the response time and recovery time of the flexile photodetector, and d) the time dependent on the on–off photocurrent response of the device before or/and after 100 cycles of bending at a voltage of 5.0 V.

In summary, an alternative facile organometallic synthetic route is developed for the growth of ultrathin Sb_2_Se_3_ nanowires with diameters ranging from 10 to 20 nm and length up to 30 μm via the reaction of triphenylantimony with dibenzyldiselenide in ethanol at 200 °C for 16 h using oleylamine and PVP as surfactants. Especially, flexible photodetectors employing ultrathin Sb_2_Se_3_ nanowires film have been firstly fabricated on PET and ordinary printing paper substrates, which demonstrate high performance. The excellent flexibility and durability of the photodetectors based on the Sb_2_Se_3_ nanowires are desirable for practical application in high‐performance flexile optoelectronic devices with low‐cost, portability and mechanical stability.

## Experimental Section

All reagents and solvents were of analytical purity and they were obtained from commercial sources and used without further purification. In a typical synthesis, 0.3 mmol (105.4 mg) triphenylantimony and 0.2 mmol (68.0 mg) dibenzyldiselenide were dissolved in 5.0 mL oleylamine and 0.2 g of PVP (Shanghai Reagent Company, *M*_W_ ≈ 30 000) was dissolved in 10 mL ethanol, respectively. Then, the two kinds of solution were mixed to form a homogenous solution that was transferred into a 25 mL Teflon‐lined stainless steel autoclave and maintained at 200 °C for 16 h. After reaction, the autoclaves were cooled down to room temperature naturally. The resulting black precipitates were washed with ethanol and hexane several times.

The as‐prepared products were identified by X‐ray powder diffraction (XRD) patterns on a Philips X′Pert Pro Super diffractometer with graphite‐monochromatized Cu Kα radiation (*λ* = 1.54178 Å). The nanowire morphologies, structure, and composition were investigated by the field emission scanning electron microscopy (SEM) (JEOL JSM‐6700F), transmission electron microscopy (TEM), and high‐resolution TEM accompanied by energy dispersive X‐ray spectroscopy on JEOL‐2010 with an accelerating voltage of 200 kV. Raman spectra were performed on a LABRAM‐HR spectroscopy with excitation of the 514.5 nm line of an Ar^+^ ion. X‐ray photoelectron spectroscopic (XPS) spectra were collected using a VGESCA‐LAB MKII X‐ray photoelectron spectrometer fitted with a monochromatic Al Kα X‐ray source (1487 eV) operating on a spot size of 560 μm. UV–vis–NIR absorption spectra were recorded on a Perkin Elmer Lambda 950 UV–vis–NIR spectrophotometer. The Fourier transformed infrared (FTIR) spectrum of the nanowires was detected by a Fourier infrared spectrometer iS10.

Flexible photodetectors based on Sb_2_Se_3_ ultrathin nanowires were fabricated on the substrates of polyethylene terephthalate (PET) and ordinary printing paper, respectively. In detail, 30.0 mg of the as‐prepared Sb_2_Se_3_ ultrathin nanowires was dispersed in 2.0 mL ethanol to make a homogeneous solution. The solution was drop‐deposited on the top side of a well‐prepared PET and printing paper plate (with size of 2.0 × 1.5 cm) by spin‐casting treatment and dried in air naturally to form an assembly layer film of the Sb_2_Se_3_ ultrathin nanowires. Afterward, ohmic contact electrode patterns (1.5 mm × 2.5 mm), Cu (5.0 nm), and Au (40.0 nm) deposition on the film by using a high‐vacuum electron beam system under the assistance of a shadow mask with spacing of 1.0 mm. The current–voltage (*I–V*) characteristics and the sensitivity of the photodetectors were measured using CHI600E electrochemical workstation, equipped with a xenon lamp of PLS‐SXE300/PLS‐SXE300UV with an output wavelength range of 400–780 nm.

## Supporting information

As a service to our authors and readers, this journal provides supporting information supplied by the authors. Such materials are peer reviewed and may be re‐organized for online delivery, but are not copy‐edited or typeset. Technical support issues arising from supporting information (other than missing files) should be addressed to the authors.

Supplementary

## References

[1] C. Liu , N. P. Dasgupta , P. D. Yang , Chem. Mater. 2014, 26, 415.

[2] J. Ye , C. Zhang , C. L. Zou , Y. L. Yan , J. M. Gu , Y. S. Zhao , J. N. Yao , Adv. Mater. 2014, 26, 620. 24357253 10.1002/adma.201303482

[3] K. Q. Peng , S. T. Lee , Adv. Mater. 2011, 23, 198. 20931630 10.1002/adma.201002410

[4] H. W. Lee , P. Muralidharan , R. Ruffo , C. M. Mari , Y. Cui , D. K. Kim , Nano Lett. 2010, 10, 3852. 20795626 10.1021/nl101047f

[5] T. Y. Zhai , L. Li , Y. Ma , M. Y. Liao , X. Wang , X. S. Fang , J. N. Yao , Y. Bando , D. Golberg , Chem. Soc. Rev. 2011, 40, 2986. 21409231 10.1039/c0cs00126k

[6] X. Fang , Y. Bando , U. K. Gautam , C. Ye , D. Golberg , J. Mater. Chem. 2008, 18, 509.

[7] R. Ulbricht , E. Hendry , J. Shan , T. F. Heinz , M. Bonn , Rev. Mod. Phys. 2011, 83, 543.

[8] S. Bai , W. W. Wu , Y. Qin , N. Y. Cui , D. J. Bayerl , X. D. Wang , Adv. Funct. Mater. 2011, 21, 4464.

[9] K. Heo , H. Lee , Y. Park , J. Park , H.‐J. Lim , D. Yoon , C. Lee , M. Kim , H. Cheong , J. Park , J. Jian , S. Hong , J. Mater. Chem. 2012, 22, 2173.

[10] R. Huang , J. Zhang , F. Wei , L. Shi , T. Kong , G. Cheng , Adv. Funct. Mater. 2014, 24, 3581.

[11] J. Ma , Y. Wang , Y. Wang , P. Peng , J. Lian , X. Duan , Z. Liu , X. Liu , Q. Chen , T. Kim , G. Yao , W. Zheng , CrystEngComm 2011, 13, 2369.

[12] R. B. Yang , J. Bachmann , E. Pippel , A. Berger , J. Woltersdorf , U. Goesele , K. Nielsch , Adv. Mater. 2009, 21, 3170.

[13] X. Wang , K. Cai , H. Liu , Cryst. Growth Des. 2011, 11, 4759.

[14] C. Zhao , X. Cao , X. Lan , Mater. Lett. 2007, 61, 5083.

[15] R. F. Service , Science 2006, 312, 1593. 16778035 10.1126/science.312.5780.1593

[16] J. A. Rogers , T. Someya , Y. Huang , Science 2010, 327, 1603. 20339064 10.1126/science.1182383

[17] Z. Wang , H. Wang , B. Liu , W. Qiu , J. Zhang , S. Ran , H. Huang , J. Xu , H. Han , D. Chen , G. Shen , ACS Nano 2011, 5, 8412. 21942659 10.1021/nn203315k

[18] B. Liu , D. Tan , X. Wang , D. Chen , G. Shen , Small 2013, 9, 1998. 23203832 10.1002/smll.201202586

[19] Y. Sun , J. A. Rogers , Adv. Mater. 2007, 19, 1897.

[20] X. Wang , W. Song , B. Liu , G. Chen , D. Chen , C. Zhou , G. Shen , Adv. Funct. Mater. 2013, 23, 1202.

[21] Y. Sun , H. H. Wang , Adv. Mater. 2007, 19, 2818.

[22] A. Manekkathodi , M. Y. Lu , C. W. Wang , L. J. Chen , Adv. Mater. 2010, 22, 4059. 20512820 10.1002/adma.201001289

[23] Y. Q. Liu , M. Zhang , F. X. Wang , G. B. Pan , J. Mater. Chem. C 2014, 2, 240.

[24] T. Zhai , M. Ye , L. Li , X. Fang , M. Liao , Y. Li , Y. Koide , Y. Bando , D. Golberg , Adv. Mater. 2010, 22, 4530. 20836072 10.1002/adma.201002097

[25] D. Choi , H. Jeong Gyoung , S. W. Kim , Y. Jang , J. Lee , D. Whang , W. Hwang Sung , K. S. Cho , S. W. Kim , Sci. Rep. 2014, 4, 6714. 25336056 10.1038/srep06714 PMC4205837

[26] R. Vadapoo , S. Krishnan , H. Yilmaz , C. Marin , Phys. Status Solidi B 2011, 248, 700.

[27] R. Jin , G. Chen , J. Pei , J. Sun , Y. Wang , Nanoscale 2011, 3, 3893. 21842088 10.1039/c1nr10584a

[28] T. Y. Ko , K. W. Sun , J. Lumin. 2009, 129, 1747.

[29] J. Wang , Z. Deng , Y. Li , Mate. Res. Bull. 2002, 37, 495.

[30] H. Kind , H. Yan , B. Messer , M. Law , P. Yang , Adv. Mater. 2002, 14, 158.

[31] S. R. Tamalampudi , Y. Y. Lu , R. Kumar U , R. Sankar , C.‐D. Liao , K. Moorthy B , C. H. Cheng , F. C. Chou , Y. T. Chen , Nano Lett. 2014, 14, 2800. 24742243 10.1021/nl500817g

[32] G. Yu , Z. Liu , X. Xie , X. Ouyang , G. Shen , J. Mater. Chem. C 2014, 2, 6104.

[33] F. X. Wang , J. Lin , W. B. Gu , Y. Q. Liu , H. D. Wu , G. B. Pan , Chem. Commun. 2013, 49, 2433. 10.1039/c3cc38996k 23411589

