# Supplementary material for: Controlled Synthesis of Ultrathin Sb2Se3 Nanowires and Application for Flexible Photodetectors
Source: Adv Sci (Weinh). 2015 Jun 25;2(10):1500109. doi: 10.1002/advs.201500109 (PMC5115312; doi:10.1002/advs.201500109)
Supplement: Supplementary file 1 — Supplementary [file ADVS-2-0l-s001.pdf]

Copyright WILEY-VCH Verlag GmbH & Co. KGaA, 69469 Weinheim, Germany, 2015.

ADVANCED SCIENCE
Open Access

Supporting Information

for *Adv. Sci.*, DOI: 10.1002/advs.201500109

Controlled Synthesis of Ultrathin Sb_2Se_3 Nanowires and Application for Flexible Photodetectors

*Guihuan Chen, Wenliang Wang, Chunde Wang, Tao Ding, and Qing Yang**

Supporting Information

Controlled Synthesis of Ultrathin Sb_2Se_3 Nanowires and Application for Flexible Photodetectors

*Guihuan Chen, Wenliang Wang, Chunde Wang, Tao Ding and Qing Yang**

G. H. Chen, W. L. Wang, C. D. Wang, T. Ding, Prof. Q. Yang
Hefei National Laboratory for Physical Sciences at Microscale (HFNL),
Department of Chemistry,
Laboratory of Nanomaterials for Energy Conversion (LNEC),
University of Science and Technology of China (USTC)
Hefei, Anhui 230026, P. R. China

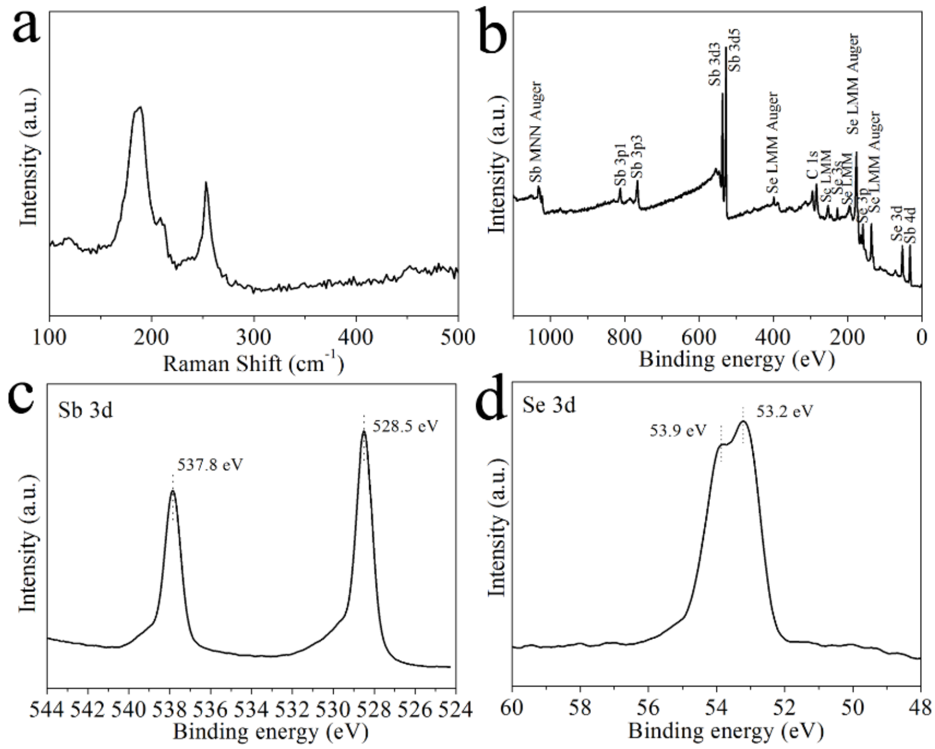

Figure S1. (a) Raman spectrum, and XPS spectra of (b) survey, (c) Sb 3d, and (d) Se 3d core levels, respectively, for the Sb_2Se_3 nanowires prepared via reaction of triphenylantimony with dibenzyldiselenide in ethanol at 200 °C for 16 h in the presence of oleylamine and polyvinylpyrrolidone (PVP).

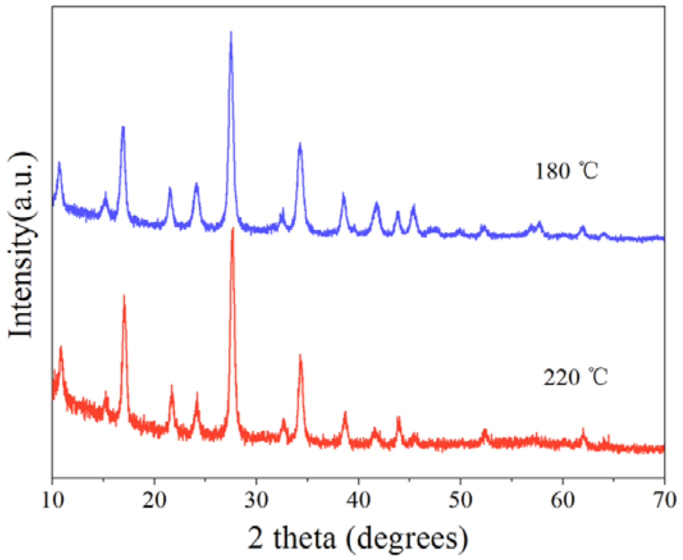

Figure S2. XRD patterns of the samples synthesized at different reaction temperature.

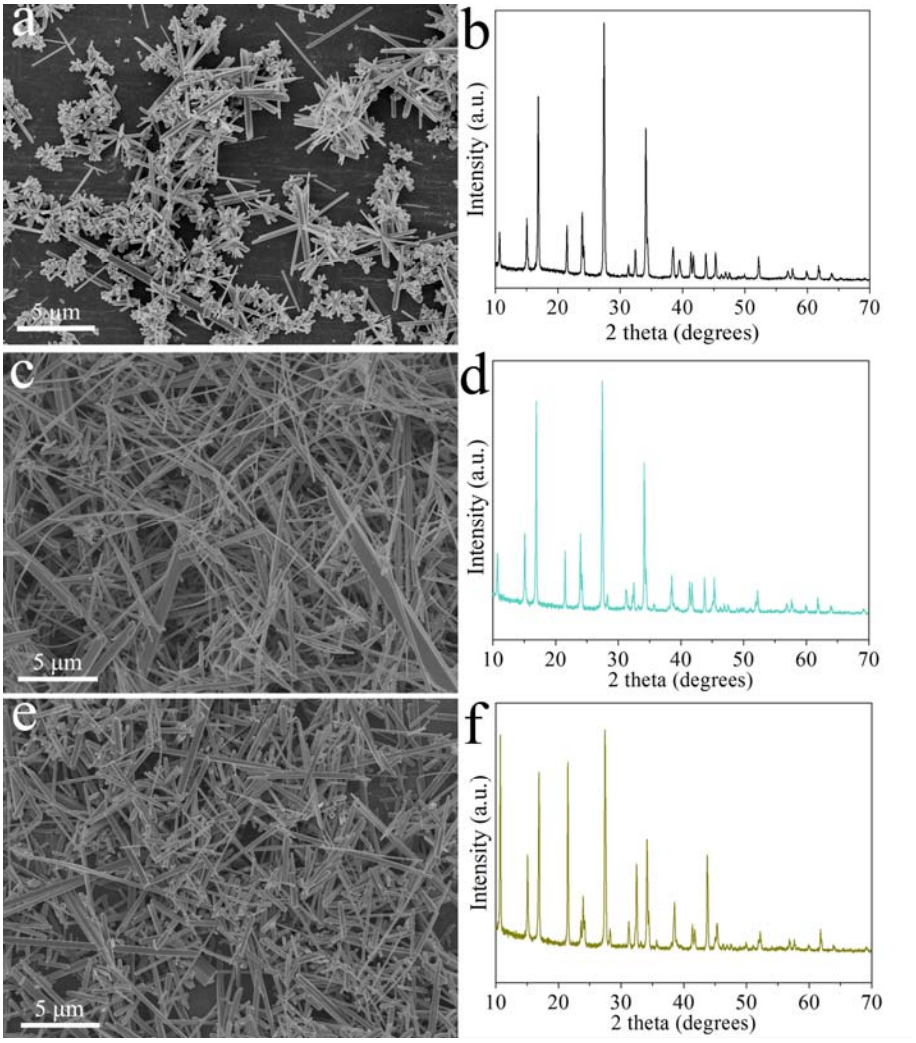

Figure S3. (a) SEM and (b) XRD pattern of the samples synthesized without PVP and oleylamine, (c) SEM and (d) XRD pattern of the samples synthesized without PVP, (e) SEM and (f) XRD pattern of the samples synthesized without oleylamine keeping

other conditions unchanging.

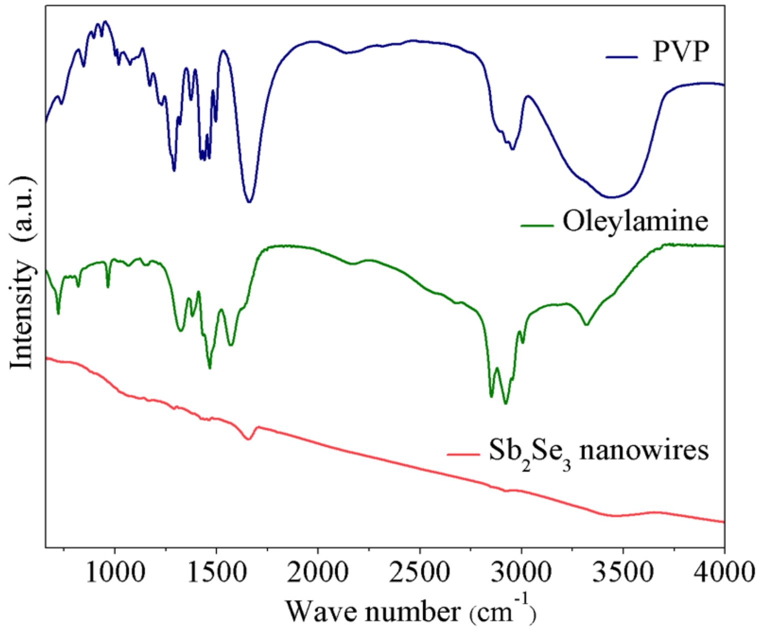

Figure S4. FTIR spectra of the PVP, oleylamine and ultrathin Sb_2Se_3 nanowires synthesized with the assistance of PVP and oleylamine, respectively.

In Figure S4, the vibration bands detect the existence of oleylamine and PVP in the final Sb_2Se_3 nanowires even though these samples have been washed thoroughly with ethanol for several times. Based on the above results, it is reasonable that oleylamine and PVP adsorbs on the surface of the nanowires (mainly on the (hk0) facets of Sb_2Se_3 based on the structure determination (Figure 1, Figure S3)), and further enhance the anisotropic formation/growth of the ultrathin nanowires.

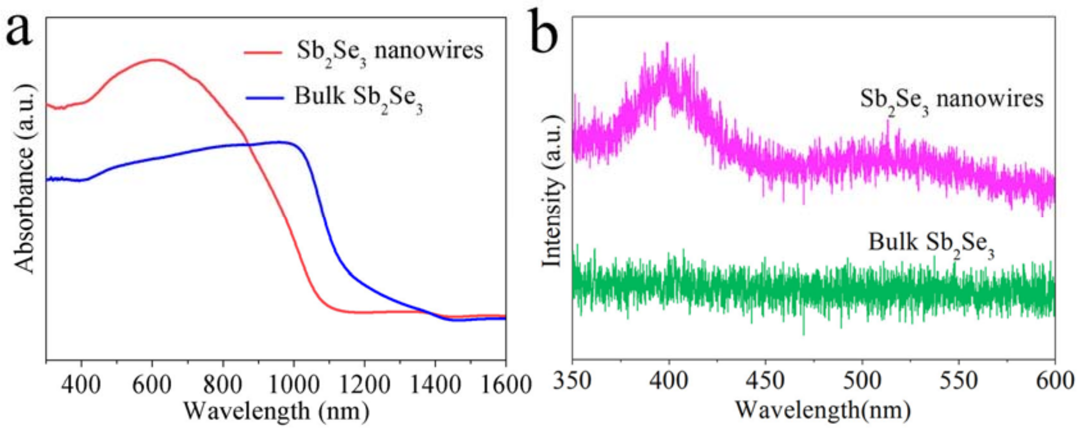

Figure S5. (a)The absorption spectra and (b) photoluminescence (PL) emission

spectra of the ultrathin Sb_2Se_3 nanowires and the bulk Sb_2Se_3, respectively.

Figure S5a is the UV-Vis spectra of the as-obtained Sb_2Se_3 nanowires and bulk Sb_2Se_3. It can be observed that the absorption band edge of the Sb_2Se_3 nanowires moves to the short wavelength comparing with the bulk Sb_2Se_3. Moreover, the photoluminescence of the ultrathin Sb_2Se_3 nanowires and bulk Sb_2Se_3 are measured. As shown in Figure S5b, with exciting of 365 nm laser, the ultrathin Sb_2Se_3 nanowires exhibit a weak fluorescence at 370-430 nm, however, there is no photoluminescence from bulk Sb_2Se_3. According to previous reports,[S1,S2] compared with that of the bulk Sb_2Se_3 crystals, the blue shift of optical absorption edge and the photoluminescence phenomena of ultrathin Sb_2Se_3 nanowires are mainly caused by the quantum confinement effect.

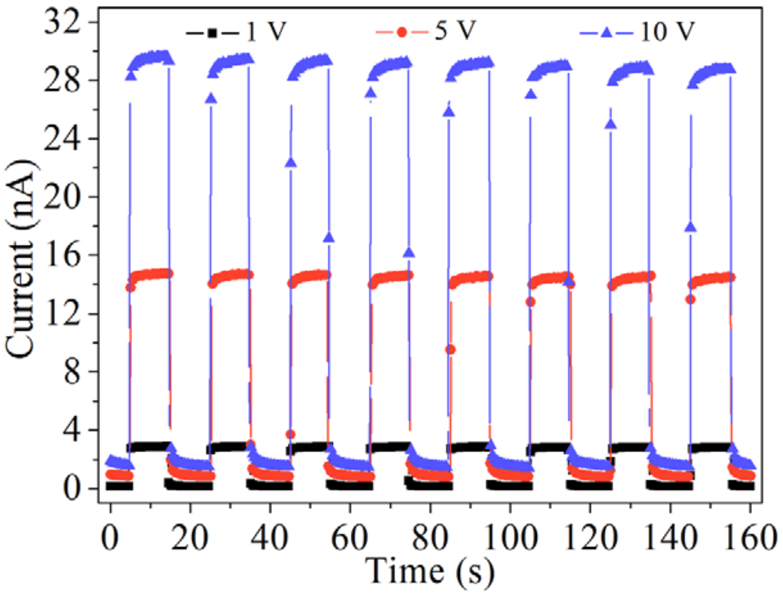

Figure S6. The time function of the on–off photocurrent response of the device (based on the PET substrate) at different voltage with a fixed light intensity of 23.8 mW/cm^2.

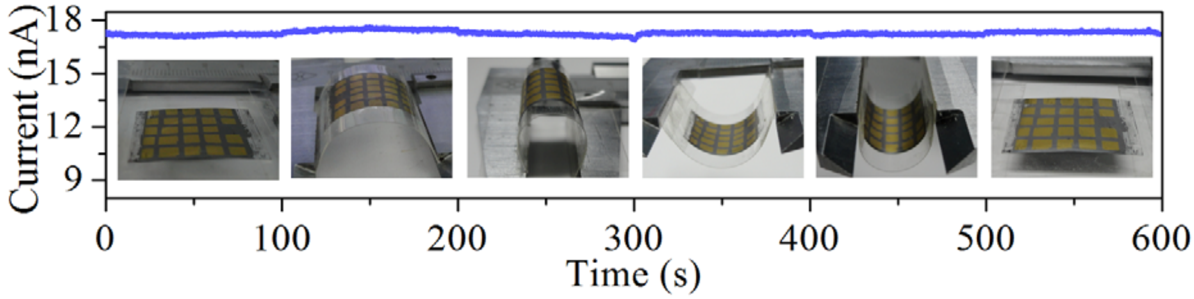

Figure S7. The I-t curve of the flexible photodetector (based on the PET substrate) when bending with different curvatures exposed to light illumination of 32.4 mW/cm^2 under a bias voltage of 5.0 V. The inserts are corresponding photographs of the device under the different bending states.

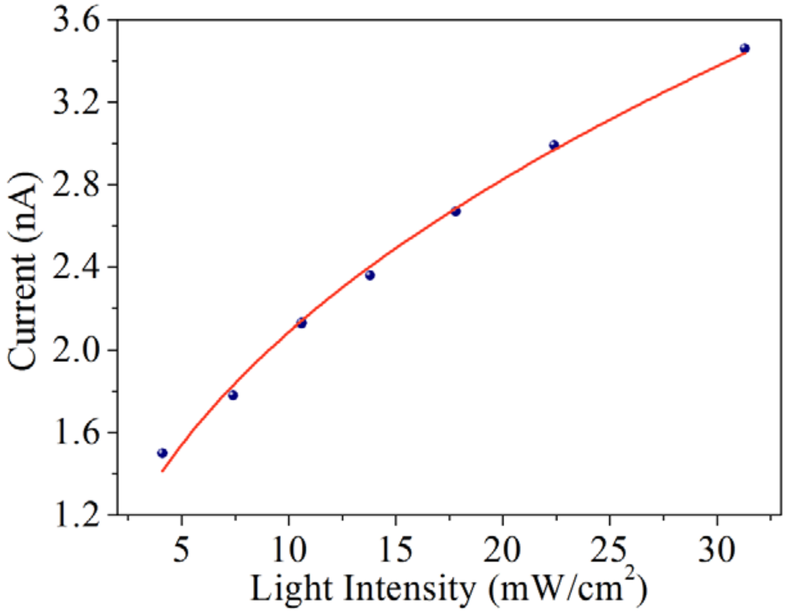

Figure S8. The photocurrent of the photodetector (based on the ordinary printing paper substrate) measured as a function of incident light density at a voltage of 5.0 V.

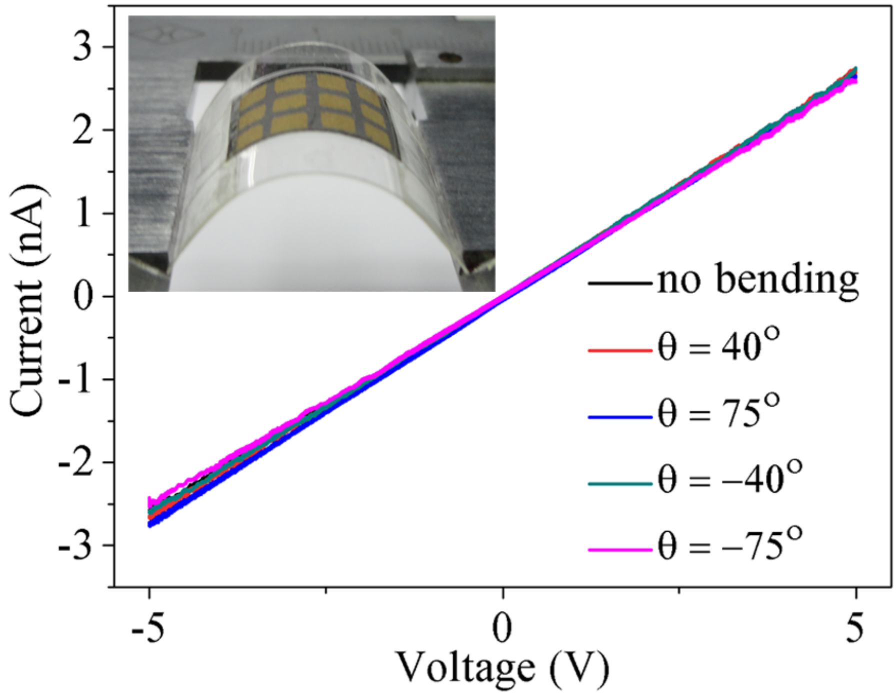

Figure S9. The I-V curves of the device based on printing paper measured at different bending angles (the positive and negative values are defined according to the folding direction of the device as demonstrated in the inset of Figure 3f). Inset: the photograph of the device.

Reference

S1. X.Y. Xiao, A.J. Fischer, G.T. Wang, P. Lu, D.D. Koleske, M.E. Coltrin, J.B. Wright, S. Liu, I. Brener, G. S. Subramania, J. Y. Tsao, *Nano Lett.* **2014**, *14*, 5616.

S2. R. J. A. Esteves, M. Q. Ho, I. U. Arachchige, *Chem. Mater.* **2015**, *27*, 1559.
